# Beyond 2015: time to reposition Scandinavia in global health?

**DOI:** 10.3402/gha.v6i0.20903

**Published:** 2013-04-03

**Authors:** Peter Byass, Peter Friberg, Yulia Blomstedt, Stig Wall

**Affiliations:** 1 Umeå Centre for Global Health Research, Umeå University, Umeå, Sweden; 2 Department of Clinical Physiology, University of Gothenberg, Gothenberg, Sweden; 3 Swedish Society of Medicine, Stockholm, Sweden

Global health currently finds itself in an exciting, almost bewildering, state of flux. A plethora of initiatives, statements, high-level meetings, and other activities are generating a continuous flow of new ideas, with the impetus at least partly driven by the advent of the 2015 target date set for the Millennium Development Goals that were adopted in 2000 (1). Whatever shape the post-2015 global health landscape may eventually take, it is already clear that there will be new targets of some kind as the world tries to make further progress on some of the less tractable health issues (2).

Against this background, the Swedish Society of Medicine (www.sls.se) has taken the initiative of organising a global health event in Stockholm from 4th to 5th April 2013. This is intended as a bottom-up framework, open to all with any kind of interest in global health, which will hear from global health practitioners, debate some of the key issues, and, most importantly, formulate a ‘Stockholm Declaration’, which will be published after the meeting. This will be a statement developed during the meeting, including the Scandinavian perspectives likely to be brought to the forum, carrying popular rather than official or institutional authority, and which will seek to achieve two aims. First, it will attempt to state where Scandinavian society stands in relation to global health and, second, define ways in which Scandinavia might prioritise engagement with the post-2015 global health landscape.

Scandinavian countries have a world-leading record when it comes to global development, at least in relation to their size, and many citizens have been directly and indirectly engaged in this. However, global health specifically is a relatively new concept, tending to supersede the former concept of international health. This somewhat changes the scenario from northern societies acting towards and for the health of southern countries to a more strongly partnership-oriented process in which global health is co-owned by the global north and south. In principle, Scandinavia should be in a strong position to make this transition: well-established principles of equity and equality combined with a lack of colonial baggage. More widely, though, it is not clear that global health has yet achieved the degree of global ownership that it needs (3), and perhaps this is one area where Scandinavian society can make a distinctive contribution in the coming years.

In terms of future priorities for global health, there are many potential agendas. It is a sad fact that the majority of the world's population and their life events remain unrecorded, even in the information-heavy 21st century, and despite Scandinavia's successful 250-year track record in this respect (4). Not surprisingly, therefore, there also remain major holes in health care provision, for which vertical disease-specific programmes have often been implemented as a poor substitute for genuinely comprehensive preventive and curative health systems. Even while the health needs of the world's population are not being effectively measured or met, those needs are themselves changing. Economics, politics, climate, and social conditions are just some of the factors which determine health alongside the direct effects of health services. At the same time, health systems can be constrained by deficiencies in knowledge, resources, and effective strategies.

**Fig. 1:**
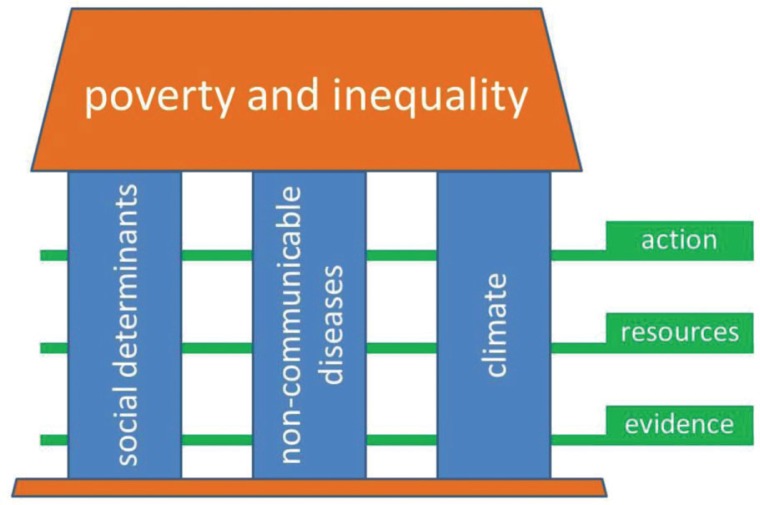
A conceptual framework covering some major aspects of global health.

For the purpose of discussions in Stockholm, we have constructed a framework representing some of the areas that we believe will be important in global health in the near future, as shown in Fig. 1. This is absolutely not intended to cover every aspect of global health, but it includes many of the major challenges. Poverty and inequality are inescapable as overarching drivers of people's health, with important global and local variations. Our three chosen pillars, social determinants, non-communicable diseases (NCD), and climate, comprise some of the major current challenges against good health. The layers of evidence, resources, and action cut across these pillars as a framework for enabling positive actions to be planned and implemented.

**Fig. 2:**
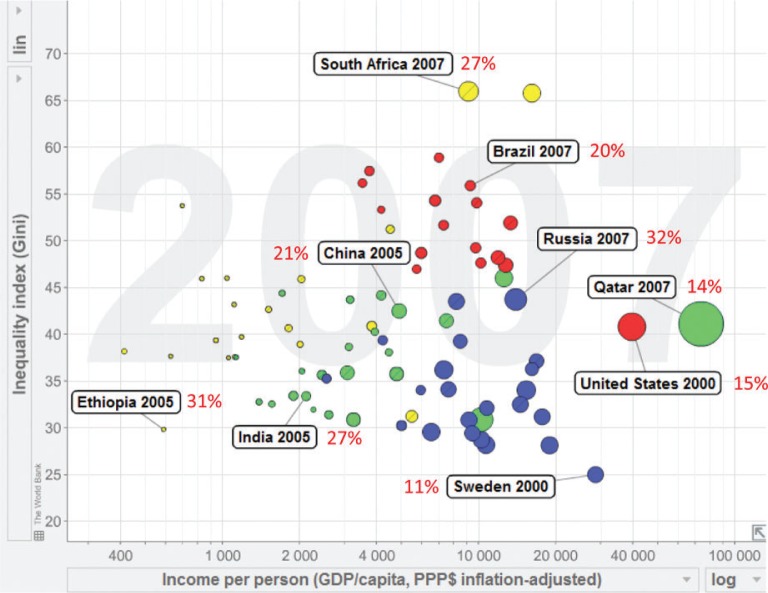
A Gapminder view of per capita income (horizontal axis), within-country inequality (vertical axis), per capita carbon emissions (represented by the size of country bubbles), and risk of dying from a NCD between 30th and 70th birthdays (red figures – data from WHO Global Health Observatory). Bubble colours are: Africa, yellow; Americas, red; Asia, green; and Europe, blue.

Figure 2 shows, in Gapminder style (www.gapminder.com), some examples of how these factors interact in the complex web of global health. The horizontal axis is a simple representation of economic status, and therefore poverty, expressed as US$ per person per year, on a national average basis. The fact that it needs to be on a logarithmic scale in order to clearly separate the various nations of the world is a damning indictment on the continuing extent of global differentials in poverty. The vertical axis adds a dimension of within-country inequality, by showing the Gini coefficient. High values reflect the most unequal societies, and low values the most equal. Then, to bring climate into play, the size of the bubbles reflects carbon emissions per person, again on a nationally averaged basis. Nine countries have been highlighted and labelled for the sake of discussion; the figures in red indicate the risk of an individual dying from a NCD between his or her 30th and 70th birthdays – representing the concept of premature NCD mortality.

Not surprisingly, the richer countries (right-hand side) are generally the greater per capita carbon emitters, but they also tend to be less affected by premature NCD mortality. This shows very clearly that premature NCD mortality is not primarily a product of affluence or industrialisation. Inequality (top area) is strongly associated with some of the world's emerging economies, which also carry substantial burdens of premature NCD mortality. Poorer, low-carbon-emitting populations, exemplified here by Ethiopia and India, also carry high burdens of premature NCD mortality.

Accordingly, in response to a call for articles, *Global Health Action* is pleased to present a series of articles which showcase examples of the various elements schematised in Fig. 1. D'Ambruoso reviews the overarching global health landscape, particularly applying an equity lens (5).

The major theme around social determinants reflects much of the thinking from the WHO Commission on the Social Determinants of Health (6). Evidence on the importance of social determinants as drivers of health is exemplified in the context of maternal health in India (7). Associated capacity development issues in the field of social determinants are reflected in the context of the EU INTREC training initiative (8). Action implications from findings on inequity in China are discussed by Yuan and colleagues (9).

NCDs are an increasingly great source of concern in global health, as a follow-up to the UN high-level meeting on this topic in September 2011 (10). NCDs also bring great complexity to global health, since they represent multiple disease entities, with various age and sex profiles and – among the oldest sectors of a population – are to some degree unavoidable. What is critically important, as reflected in a resolution of the World Health Assembly in 2012, is to control and reduce burdens of premature NCD mortality and associated morbidity. Paddick and colleagues discuss approaches to revealing the burden of dementia in Africa (11). Training community health workers to handle new demands of NCDs is tackled in the 5-SPICE framework (12), and approaches to acting against tobacco in Nigeria have been set out by Oladele and colleagues (13).

Climate change represents an increasing challenge to human health (14). Weather conditions have always affected health, and it is important to learn from the long-term health consequences of meteorological conditions, as presented from historic Swedish data (15), in terms of understanding what the health effects of future hypothetical climate change might be. Climate and health covers a particularly wide range of stakeholders, and Stordalen and colleagues discuss the need for better integration between academia, enterprise, governments, and international bodies (16) to effectively protect populations from detrimental effects of climate. Kjellstrom and McMichael discuss strategies for responding to climate threats, with a particular focus on the effects of increased heat (17).

This set of articles certainly does not answer all the questions about patterns of global health problems and solutions for the post-2015 era. However, they do represent a broad-based selection of case studies with truly global representation. They are presented as a resource both for the 2013 meeting in Stockholm and for the ensuing ‘Stockholm Declaration’.

*Peter Byass* Umeå Centre for Global Health Research Umeå University, Umeå Sweden *Peter Friberg* Department of Clinical Physiology University of Gothenberg, Gothenberg Sweden Swedish Society of Medicine, Stockholm Sweden *Yulia Blomstedt* Umeå Centre for Global Health Research Umeå University, Umeå Sweden *Stig Wall* Umeå Centre for Global Health Research Umeå University, Umeå Sweden
